# Acceptability of a Korean Medicine-Based Obesity Program Among Patients Achieving Predefined Weight-Loss Targets: A Retrospective Mixed-Methods Study

**DOI:** 10.3390/nu18172937

**Published:** 2026-09-07

**Authors:** Ayusha Pandey, Dasol Park, Suyong Shin, Jungsang Kim, Minwhee Kang, Donghun Lee, Junho Kim, Chunghee Kim, Jiyoung Son, Seonghyeon Jeon, Minwoo Bang, Byungsoo Kang, Jungtae Leem

**Affiliations:** 1 Department of Digital Healthcare, Graduate School of Jeonbuk Advanced Bioconvergence Academy, Wonkwang University, 460, Iksan-daero, Iksan-si 54538, Jeonbuk-do, Republic of Korea; ayushapandey232@gmail.com; 2 Department of Diagnostics, College of Korean Medicine, Wonkwang University, 460, Iksan-daero, Iksan-si 54538, Jeonbuk-do, Republic of Korea; mare927@naver.com; 3 Daeat Korean Medicine Clinic Incheonbupyeong, 17, Jubuto-ro, Bupyeong-gu, Incheon 21390, Republic of Korea; sypride081@daeat-bp.com; 4 Department of Preventive Medicine, College of Korean Medicine, Kyung Hee University, 26, Kyungheedae-ro, Dongdaemun-gu, Seoul 02447, Republic of Korea; 5 Daeat Korean Medicine Clinic Gyeonggisuwon, 141, Jungbu-daero, Paldal-gu, Suwon-si 16240, Gyeonggi-do, Republic of Korea; sportplayer1@daeat-sw.com; 6 Daeat Korean Medicine Clinic Gyeonggiilsan, 33, Jeongbalsan-ro, Ilsandong-gu, Goyang-si 10402, Gyeonggi-do, Republic of Korea; kmh@daeat-is.co.kr; 7 Daeat Korean Medicine Clinic Busan, 177, Beomil-ro, Busanjin-gu, Busan 47358, Republic of Korea; dh.lee@daeat-bs.co.kr; 8 Daeat Korean Medicine Clinic Daegu, 341, Dongdaegu-ro, Suseong-gu, Daegu 42019, Republic of Korea; dgdaeat@daeat-dg.co.kr; 9 Daeat Korean Medicine Clinic Changwon, 103, Jungdongjungang-ro, Uichang-gu, Changwon-si 51380, Gyeongnam-do, Republic of Korea; megachung1@daeat-cw.co.kr; 10 Daeat Research Institute, 803, Seolleung-ro, Gangnam-gu, Seoul 06019, Republic of Korea; sjy0425@daeat-ca.co.kr; 11 Daeat Korean Medicine Clinic Cheonan, 73-8, Buldang 23-ro, Seobuk-gu, Cheonan-si 31156, Chungnam-do, Republic of Korea; 12 Daeat Korean Medicine Clinic Daejeon, 56, Dunsan-ro, Seo-gu, Daejeon 35226, Republic of Korea; rastgeleo@daeat-dn.co.kr; 13 Daeat Korean Medicine Clinic Seoul, 803, Seolleung-ro, Gangnam-gu, Seoul 06019, Republic of Korea; 14 Department of Ophthalmology, Otolaryngology & Dermatology, College of Korean Medicine, Gachon University, 1342, Seongnam-daero, Sujeong-gu, Seongnam-si 13120, Gyeonggi-do, Republic of Korea; 15 Department of Il-Won Integrated Medicine, Wonkwang University Korean Medicine Hospital, 895, Muwang-ro, Iksan-si 54538, Jeonbuk-do, Republic of Korea; 16 Research Center of Traditional Korean Medicine, Wonkwang University, 460, Iksan-daero, Iksan-si 54538, Jeonbuk-do, Republic of Korea

**Keywords:** obesity, Korean medicine, patient acceptability, retrospective chart review, mixed-methods research

## Abstract

**Background and Objectives**: Although herbal medicine-based obesity treatment programs are used in obesity management, empirical research on patient acceptability remains limited. This study examined acceptability of a Korean medicine-based obesity treatment program (KM-OBP) among patients who achieved predefined weight-loss targets in routine clinical care. **Materials and Methods**: Medical records and satisfaction survey data routinely collected during clinical practice at a single Korean medicine clinic were retrospectively analyzed. Patients had baseline body mass index (BMI) ≥ 30 kg/m^2^ and achieved both ≥10% weight loss and BMI ≤ 23 kg/m^2^ during follow-up. BMI trajectories and adverse-event occurrence were summarized descriptively. Satisfaction ratings were reported on a 5-point Likert scale, and free-text responses were retrospectively analyzed using the Theoretical Framework of Acceptability. **Results**: Of 3798 patients with a baseline BMI ≥ 30 kg/m^2^ and at least two BMI measurements, 37 (0.97%) met the predefined targets; 16 completed the satisfaction survey. Mean BMI change from the first to last measurement was −8.9 kg/m^2^, and median change was −8.8 kg/m^2^ (interquartile range, −9.6 to −7.7). Common documented adverse events were constipation, nausea, and dizziness. Satisfaction ratings were descriptively high for the overall program, perceived helpfulness for obesity treatment, and herbal medicine. Among the 16 survey respondents, perceived weight-loss benefits, program structure, safety, and health improvement were described as facilitators of acceptability, whereas post-treatment weight regain, dietary burden, and financial concerns were described as barriers. **Conclusions**: The findings characterize satisfaction and reported acceptability experiences among survey respondents within a highly selected target-achieving cohort. Interpretation is limited by outcome-based selection, potential non-response bias, the small survey sample, and the single-center design.

## 1. Introduction

Obesity is a chronic and relapsing disease that increases the future burden of non-communicable diseases, including diabetes mellitus, cardiovascular diseases, and cancer, and its prevalence continues to rise across all countries [1,2]. In 2021, overweight and obesity were estimated to be attributable to 3.71 million deaths and 129 million disability-adjusted life-years worldwide, and if current historical trends persist, approximately half of the global adult population is projected to be living with overweight or obesity by 2050 [1,3]. South Korea has followed a similar trajectory, with the prevalence of adult obesity increasing from 30.2% in 2012 to 38.4% in 2021, representing a 1.27-fold increase. Notably, the prevalence of severe obesity has risen markedly, contributing to an increasing obesity-related healthcare burden [4].

Current clinical guidelines recommend dietary interventions, increased physical activity, and behavioral interventions as first-line treatments for obesity, with pharmacotherapy and bariatric surgery considered adjunctive options [5]. Commonly used anti-obesity medications include orlistat, naltrexone/bupropion, liraglutide, and phentermine/topiramate, while bariatric surgery is the most frequently used surgical approach [6]. However, lifestyle interventions and pharmacotherapy are often associated with weight regain after treatment discontinuation [7,8], and pharmacotherapy is further limited by drug-specific adverse effects, including gastrointestinal symptoms [9,10,11], sympathetic nervous system activation, sensory and cognitive disturbances [12], and teratogenic risks during pregnancy [13], resulting in treatment restrictions and high discontinuation rates [14]. Although bariatric surgery provides substantial short- and long-term weight loss and is relatively safe when performed by experienced clinicians, it remains invasive, with potential surgical complications, limited eligibility, and the need for long-term management of adverse effects [15]. Taken together, these limitations underscore the need for complementary and alternative multimodal approaches to obesity management.

Several studies have reported that herbal medicine-based treatments safely improve body weight and obesity-related indicators in patients with obesity [16,17,18,19]. Herbal medicine-based obesity treatment programs are typically designed with multiple components rather than herbal medicine alone. In this regard, a survey study of Korean medicine (KM) doctors examining obesity treatment programs reported that patient satisfaction varied substantially across treatment components, including herbal medicine, dietary modification, and pharmacopuncture [20]. Additionally, Kuzmar et al. reported that visual confirmation of outcomes, such as reductions in waist circumference, was associated with lower dropout rates during the program [21]. These findings suggest that treatment continuation may be influenced not only by the effectiveness and safety of herbal medicine but also by the design and composition of the overall program. Accordingly, there is a need for research examining the factors that influence patient acceptability of obesity treatment programs. However, empirical evidence on the acceptability of herbal medicine-centered obesity treatment programs remains limited.

A previous single-center retrospective observational study examined clinical outcomes and safety of a Korean medicine-based obesity program (KM-OBP) incorporating herbal medicine and lifestyle modification [18]; however, patient acceptability and the experiential factors reported by participants were not specifically investigated.

Accordingly, this study aimed to explore acceptability of a KM-OBP with herbal medicine as its core component specifically among patients who achieved predefined weight-loss and BMI targets in routine clinical care. Using a retrospective mixed-methods approach based on routinely collected records and survey data, BMI trajectories and adverse-event occurrence were presented as descriptive clinical context, while satisfaction ratings and free-text responses were used to characterize the experiences reported by survey respondents.

## 2. Methods

### 2.1. Study Design and Participants

A retrospective convergent mixed-methods approach [22] was used to describe the clinical context of patients who achieved predefined targets and to examine satisfaction and acceptability-related experiences among survey respondents within this cohort. The study was conducted in a real-world outpatient setting at a single Korean medicine clinic. Herbal prescriptions, optional adjunctive therapies, treatment duration, and follow-up were determined by attending Korean medicine physicians during routine care according to patients’ clinical conditions and preferences rather than being predetermined for research purposes; clinical data were retrospectively obtained from records generated during care. Quantitative satisfaction ratings and qualitative free-text responses were analyzed as complementary strands of equal priority and integrated at the interpretation stage.

Patients were eligible if they were new patients between July 2021 and December 2023, had baseline BMI ≥ 30 kg/m^2^, received a KM-OBP including herbal medicine, and met the prespecified criteria of both ≥10% weight loss and BMI ≤ 23 kg/m^2^ during follow-up through 31 October 2025. A ≥10% reduction was retained as an established benchmark of successful weight loss [23], and BMI ≤ 23 kg/m^2^ was selected with reference to the lower BMI threshold associated with increased health risk in Asian populations [6,24]. Because all patients with baseline BMI ≥ 30 kg/m^2^ who reached BMI ≤ 23 kg/m^2^ also exceeded 10% weight loss, the ≥10% criterion was operationally redundant but was retained as part of the prespecified definition. This author-defined subgroup is described as target-achieving patients rather than as an established clinical category. Patients lacking pre- or post-treatment BMI or receiving concurrent conventional weight-loss interventions were excluded. The 37 eligible patients formed the clinical cohort; the 16 who completed the survey formed the patient-reported subset.

The KM-OBP implemented at Daeat KM Clinic [18] primarily consisted of herbal medicine and nutritional counseling with regular monitoring (Appendix A.1). During the first three days after program initiation (initial 3-day program [25]), patients are instructed to refrain from consuming regular meals and instead consume meal replacements containing complex carbohydrates and plant-based protein, along with herbal medicine intended to suppress appetite and support gastrointestinal function and bowel movements (Table A1). Throughout the program, herbal medicine prescriptions are determined by the attending KM physician based on each patient’s clinical characteristics and formulation preferences (Table A2). Nutritional counseling is primarily delivered by recommending a low-carbohydrate diet, and patients experiencing significant hunger are advised to consume small amounts of high-protein foods, such as boiled eggs or tofu. Adequate fluid intake, avoidance of excessive potassium intake, and maintenance of light, routine physical activity are encouraged. Concomitant use of agents with diuretic or laxative effects is discouraged, and patients are instructed to report any adverse events to the medical staff promptly. Monitoring is conducted at approximately weekly intervals, during which adverse events occurring during the KM-OBP and adherence to dietary recommendations are assessed and additional nutritional counseling is provided. Additional external therapies are selectively applied on an individual basis. When indicated, optional modalities, including pharmacopuncture, thread embedding therapy, and radiofrequency treatment applied to the abdomen, upper extremities, and lower extremities, are administered according to individual patient needs. Accordingly, the KM-OBP was a heterogeneous multimodal clinical program in which exposure to specific herbal formulations and optional adjunctive modalities varied across patients.

### 2.2. Data Collection

Data were collected to capture both the clinical context of the KM-OBP and patient-reported experiences related to program acceptability. Clinical data were extracted from routinely collected medical records. Baseline characteristics recorded at the initial visit included age, sex, alcohol consumption (binary), prior weight-loss treatment experience (either conventional medicine or KM), concomitant medication use, concomitant symptoms, past medical history, and family history. Concomitant symptoms assessed included sleep disturbance, peripheral edema, and difficulty with bowel movements. Information on the components of the KM-OBP delivered to each patient was extracted from medical records, along with longitudinal clinical course data, including serial BMI measurements during the program and the occurrence of any adverse events. For each adverse-event category, a patient was counted when at least one occurrence was documented during treatment, and an individual patient could contribute to more than one category. Adverse-event severity was not formally graded because standardized severity assessments were unavailable in the retrospective records.

Patient-reported quantitative data were obtained from a satisfaction survey routinely collected for clinic quality improvement from patients who voluntarily agreed to participate. The survey was not developed or validated as an acceptability instrument; therefore, quantitative results were interpreted as satisfaction ratings and related perceptions. Optional free-text responses were subsequently analyzed in relation to acceptability. In addition to seven explanatory free-text items paired with rating questions in Table 1, two treatment-choice prompts asked why respondents chose Korean medicine and why they chose the Daeat Clinic. Written informed consent was obtained before survey participation. For survey respondents, treatment duration was obtained from clinical records and denoted documented participation in the KM-OBP, as in Table 2. Survey timing was calculated as the interval from program initiation to survey completion. Because this interval was skewed, it was summarized using the median, interquartile range, and range.

Use of medical record data was conducted retrospectively with anonymization, and the requirement for individual consent was waived. For the satisfaction survey, written informed consent was obtained from all respondents prior to participation.

### 2.3. Data Analysis

Descriptive statistics summarized demographic, clinical, and survey data. Continuous variables were reported as means with standard deviations (SDs), medians with interquartile ranges (IQRs), and ranges; categorical variables were reported as frequencies and percentages. BMI trajectories and change from the first to the last available BMI measurement were treated as descriptive clinical context, and no inferential test of BMI change was performed. Survey respondents and non-respondents were compared descriptively using standardized mean differences (SMDs), without hypothesis testing. There were no missing clinical data for variables included in the analyses and no missing responses for applicable closed-ended survey items; no imputation was performed. Patients not exposed to an optional modality were classified as not applicable and excluded from that item’s denominator. Analyses were performed using RStudio (v.2025.9.2.418; Posit Software, PBC, Boston, MA, USA).

Qualitative content analysis [26] was conducted on free-text survey responses. The Theoretical Framework of Acceptability (TFA) [27] was introduced retrospectively as the analytical framework and did not inform the original survey design. All 16 respondents provided at least one free-text response. Across seven explanatory and two treatment-choice prompts, 107 responses (140 sentences; 931 words) were analyzed. A predefined codebook covered affective attitude, burden, ethicality, intervention coherence, opportunity costs, perceived effectiveness, and self-efficacy. A single response could receive multiple codes.

Two researchers (DP and JL) independently coded the responses after pilot coding. Codebook revisions were reapplied to previously coded material. The researchers met weekly to resolve discrepancies by consensus; unresolved cases were adjudicated by a third researcher (BK). DP, JL, and BK were not involved in participants’ clinical care or in the development or delivery of the KM-OBP. Because coding and codebook refinement were iterative and consensus-based, no formal inter-coder agreement coefficient was calculated. Within each TFA domain, similar codes were grouped into subthemes and related subthemes into broader themes; labels and boundaries were iteratively checked against the source responses. Quotations were selected to represent the breadth of experiences rather than the most detailed respondents. Data saturation was not formally assessed because the dataset comprised a fixed set of brief, retrospectively available survey responses rather than prospectively sampled interviews.

After the quantitative and qualitative analyses were completed separately, the findings were integrated at the interpretation stage. Overall and component-specific satisfaction patterns were compared with corresponding qualitative themes and subthemes to identify convergence, complementarity, or discordance. Qualitative findings were used to explain or contextualize quantitative patterns, and the integrated findings informed the interpretation of patient acceptability.

### 2.4. Ethical Considerations

For the satisfaction survey, written informed consent was obtained from all respondents prior to participation. Use of medical record data was conducted retrospectively with anonymization, and the requirement for individual consent was waived. This study protocol was approved by the Daeat Clinic Institutional Review Board on 8 January 2026 (DIRB-202601-08).

## 3. Results

**Figure 1:**
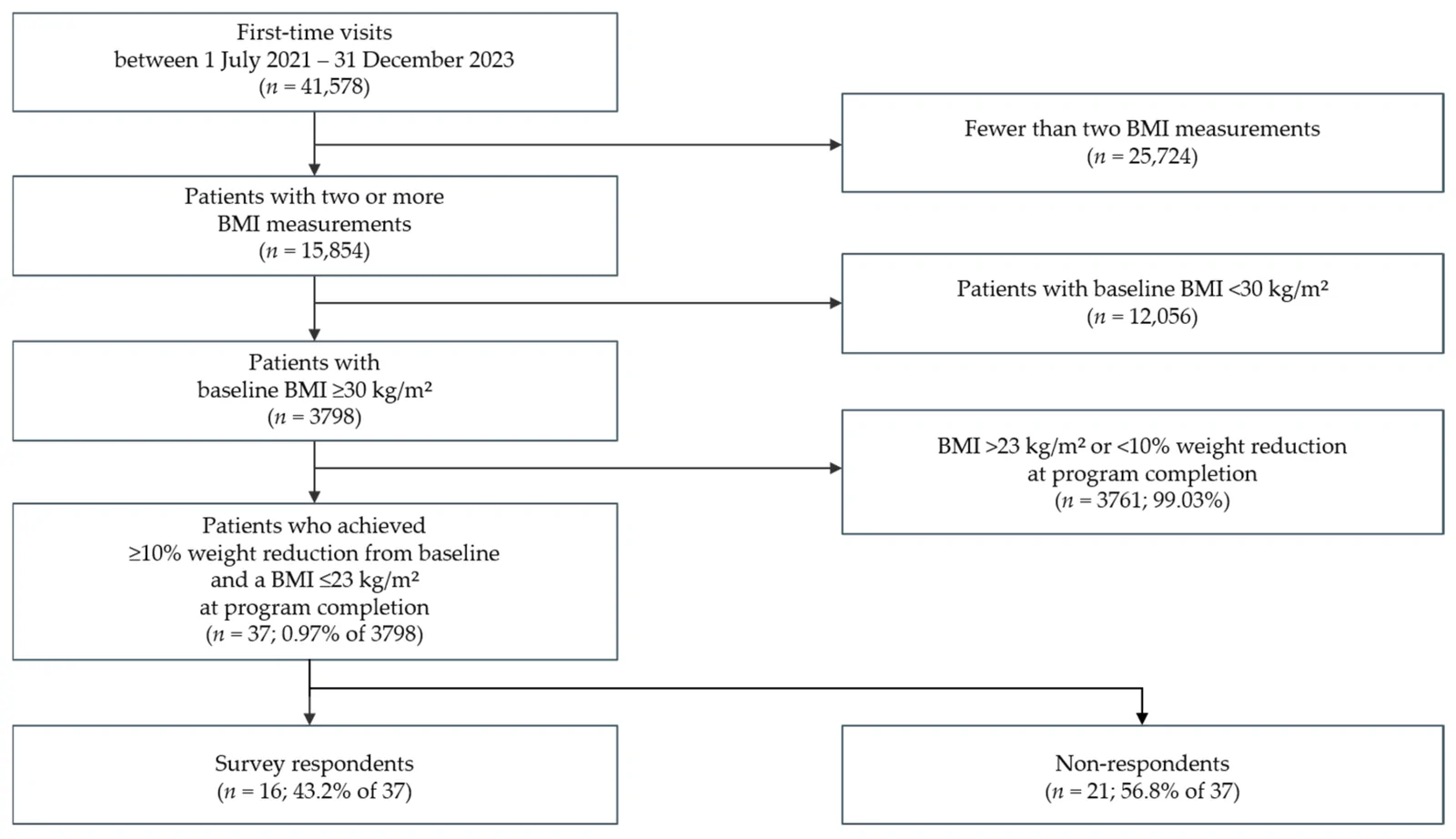
Flow diagram of patient selection. Denominators, reasons for exclusion, and survey participation are shown.

Between July 2021 and December 2023, 41,578 patients made a first visit; 15,854 had at least two BMI measurements, and 3798 had baseline BMI ≥ 30 kg/m^2^. Of these 3798 patients, 37 (0.97%) achieved both ≥10% weight loss and BMI ≤ 23 kg/m^2^ during follow-up and formed the target-achieving clinical cohort. Sixteen of the 37 patients completed the satisfaction survey (43.2%) and formed the patient-reported subset, whereas 21 (56.8%) were non-respondents (Figure 1).

**Figure A1:**
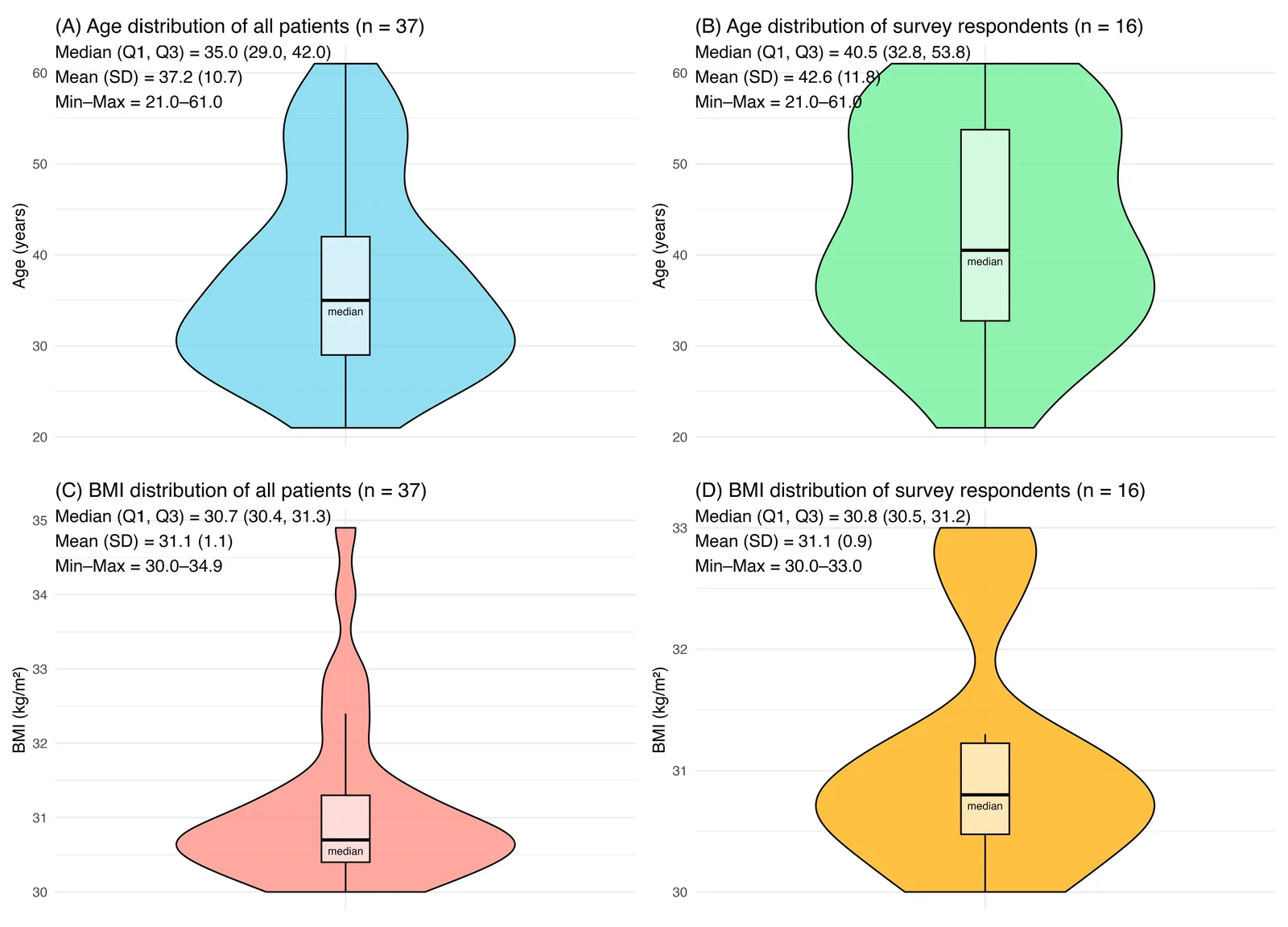
Age and body mass index (BMI) distributions of included patients. (**A**) Age distribution of all patients. (**B**) Age distribution of survey respondents. (**C**) BMI distribution of all patients. (**D**) BMI distribution of survey respondents.

Clinical characteristics of all 37 patients, 16 survey respondents, and 21 non-respondents are summarized in Table 2. Respondents were older, had longer documented treatment duration, and more often had at least one documented adverse event than non-respondents; the corresponding SMDs were 0.95, 0.67, and 0.83. Baseline BMI and BMI change were descriptively similar between the survey respondents and non-respondents, with corresponding SMDs of −0.09 and 0.18, respectively. Age and baseline BMI distributions are shown in Figure A1.

**Figure 2:**
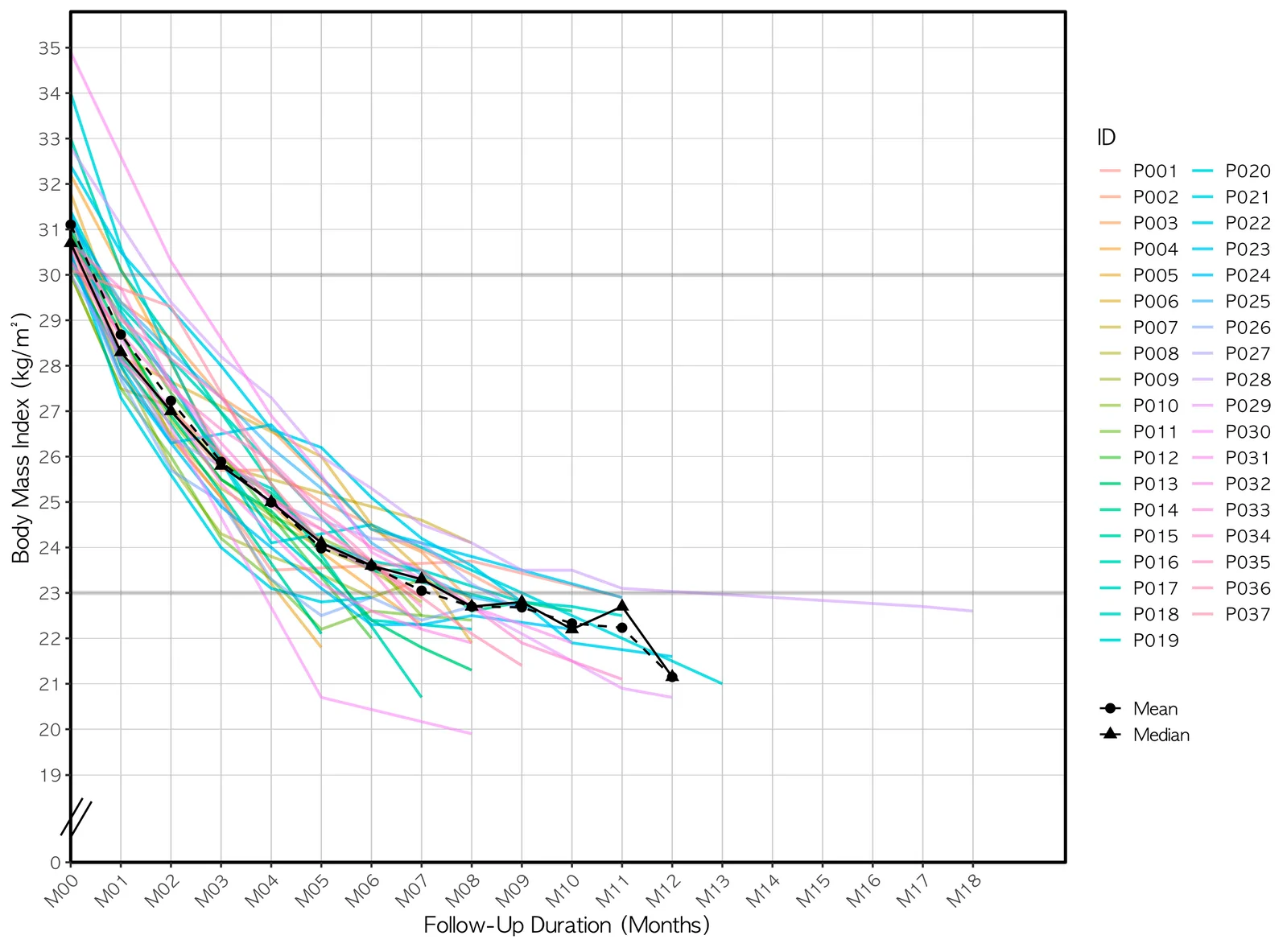
Body mass index trajectories of all 37 target-achieving patients. Circles and triangles indicate the mean and median, respectively, among patients with an available BMI measurement at each month; the number contributing therefore decreases over time. The gray horizontal lines indicate BMI thresholds of 30 and 23 kg/m^2^, corresponding to the predefined baseline eligibility and target-achievement criteria, respectively.

BMI trajectories for the 37 target-achieving patients are shown in Figure 2 as descriptive clinical context. Treatment duration, defined as actual participation in the KM-OBP, ranged from 6 to 35 months. Because some care was delivered remotely, BMI was not measured throughout the entire treatment period. The BMI measurement period, defined from baseline to the last available in-person BMI measurement and represented in Figure 2, ranged from 5 to 18 months. Time to target BMI, defined from baseline to the first BMI ≤ 23 kg/m^2^, ranged from 5 to 17 months. The first-to-last BMI change averaged −8.9 (1.6) kg/m^2^; the median was −8.8 kg/m^2^ (IQR, −9.6 to −7.7; range, −13.0 to −5.9). These changes were not tested inferentially because outcome achievement defined cohort eligibility.

Documented adverse events included constipation in 20 patients (54.1%), nausea in 7 (18.9%), and dizziness in 7 (18.9%) in the full cohort. Corresponding values among survey respondents were 12 (75.0%), 6 (37.5%), and 4 (25.0%), respectively (Table A3). These values represent patients with at least one documented occurrence; severity was not formally graded.

Satisfaction ratings are summarized descriptively in Table A4. Overall program satisfaction, perceived helpfulness for obesity treatment, and herbal medicine each had a mean score of at least 4.5. Item-level denominators ranged from 7 to 16 because patients not exposed to an optional modality were excluded as not applicable; the full response distributions are reported to avoid relying on mean differences between small, unequal denominators.

Among the 16 survey respondents, mean treatment duration was 15.1 months (SD, 5.0), and the median was 13.5 months (IQR, 12.0–19.3). Surveys were completed a median of 16.7 months after program initiation (IQR, 13.2–18.7; range, 9.6–54.5). All respondents completed the survey after target achievement and documented treatment completion.

All 16 survey respondents contributed at least one free-text response. Across the 107 responses, participants described successful weight loss, perceived health improvement, structured program delivery, professional expertise, perceived safety, responsive monitoring, and improved understanding of diet and metabolism as facilitators of acceptability. These accounts reflected positive affective attitudes, ethicality, intervention coherence, perceived effectiveness, and self-efficacy within the TFA (Table 3 and Table A5).

Participants also described financial burden, carbohydrate restriction, difficulty maintaining dietary changes, weight regain after program completion, perceived lack of proactive support, and uncertainty about optional adjunctive modalities as barriers or negative experiences. Most TFA domains were represented by multiple respondents; opportunity costs were represented by two respondents and two coding units and should still be interpreted cautiously because of the limited contribution (Table A5).

The integration of descriptive satisfaction ratings with corresponding qualitative findings is summarized in Table 4. No clear discordant patterns were identified across the quantitative and qualitative findings.

## 4. Discussion

### 4.1. Summary of Findings

This retrospective mixed-methods study described clinical trajectories in 37 patients who achieved predefined targets and examined satisfaction and acceptability-related experiences among the 16 survey respondents within that cohort. Satisfaction ratings were descriptively high for the overall program and herbal medicine, while free-text responses described perceived weight-loss benefits, program structure, safety, and health improvement as facilitators and weight regain, dietary burden, and financial concerns as barriers. These findings characterize the selected respondents’ perceptions, although links to treatment engagement or weight loss remains unclear.

### 4.2. Acceptability of Korean Medicine-Based Obesity Treatment Programs

Obesity is a chronic, relapsing disease influenced by multiple factors, and in its treatment, achieving effective and safe weight loss as well as preventing weight regain after initial success remain major challenges. Maintenance of a healthy lifestyle is recommended as a first-line approach, but achieving and sustaining weight loss through lifestyle modification alone is often challenging [7,28]. Conventional anti-obesity medications may also be followed by weight regain after discontinuation, and adverse effects can complicate long-term use [29]. In this context, some KM-OBPs, which primarily incorporate herbal medicine, have shown favorable short-term weight-loss and safety findings; however, available evidence does not establish their comparative or long-term effectiveness and safety. KM-OBPs vary from herbal-medicine-only interventions to programs incorporating dietary modification and exercise, so patient experiences and treatment demands may differ substantially across programs [30]. Accordingly, patients’ experiences with and responses to the overall program may be relevant to treatment continuation and post-program weight management, although these relationships were not examined in the present study and should be considered separately from demonstrated clinical effectiveness and safety.

A national survey of Korean medicine doctors reported markedly higher patient satisfaction with herbal medicine than with pharmacopuncture [20]. In our small, outcome-selected survey subset, descriptive ratings were also higher for herbal medicine, while free-text responses included both favorable and uncertain perceptions of optional external therapies. Prior studies examining dietary counseling and monitoring have reported variable dropout rates despite favorable weight-loss outcomes and minor adverse events [30], and randomized trials have shown lower dropout rates when herbal medicine was combined with lifestyle education than with herbal medicine alone [16,17]. In addition, interventions incorporating regular feedback and motivation have been associated with reduced dropout [21]. Consistent with these findings, our qualitative analysis showed that participants described the perceived effectiveness of herbal medicine and structured monitoring and feedback as positive experiences, whereas dietary demands and post-program weight regain were described as negative experiences. These observations contextualize acceptability across program components but do not demonstrate that any component caused treatment continuation or weight-loss outcomes. They also suggest that program design is relevant to acceptability and that more comprehensive exploration, including patients who discontinue treatment or do not reach the predefined targets, is warranted.

### 4.3. Limitations, Strengths and Implications for Future Research

This study has several important limitations. First, outcome-based selection excluded patients who discontinued treatment or did not meet the predefined targets, so the findings cannot characterize KM-OBP acceptability in the broader treated population. Second, only 16 of 37 eligible patients completed the survey. Respondents were older, had longer treatment duration, and more often had documented adverse events than non-respondents, indicating potential non-response bias. Third, 36 of 37 patients were women, and the single-clinic setting further limits generalizability. Fourth, the satisfaction survey was designed for quality improvement rather than validated acceptability assessment, and its optional brief free-text responses provided less depth than dedicated interviews; saturation was not formally assessed. The timing of survey completion after treatment varied across respondents; the interval from program initiation to survey completion ranged from 9.6 to 54.5 months. Consequently, responses, particularly those concerning post-treatment weight regain, may reflect different durations of post-treatment experience and may be affected by recall or temporal response bias. Fifth, herbal prescriptions and optional modalities varied across patients, and standardized longitudinal measures of diet, physical activity, stress, sleep, and adherence were unavailable. The small outcome-selected sample could not support a valid multivariable covariate model without severe overfitting. Finally, ingredient exposure was not standardized and constituent-specific outcomes were not assessed; findings should therefore be interpreted at the overall-program and component levels rather than in relation to individual supplements or bioactive constituents.

Despite these limitations, the study combines descriptive clinical context with quantitative satisfaction ratings and TFA-guided analysis of free-text survey responses. This approach identifies the range of facilitators, barriers, and experiential considerations reported by a selected group of target-achieving survey respondents and may inform questions and measurement priorities for future studies.

Future studies should prospectively assess acceptability across the full treatment trajectory, including patients who discontinue treatment or do not meet weight-loss targets. Multicenter studies should use validated acceptability instruments and prespecify longitudinal measurement of program exposure, dietary adherence, physical activity, stress, sleep, and post-treatment follow-up to evaluate how patient experiences relate to sustained participation without conflating perceived effectiveness with demonstrated clinical effectiveness.

## 5. Conclusions

Among the 16 survey respondents within a cohort of 37 patients who achieved predefined weight-loss targets, participants described perceived weight-loss benefits, structured program delivery, safety, and health improvement as facilitators of acceptability, and post-treatment weight regain, dietary burden, and financial concerns as barriers. These findings characterize experiences in a highly selected subgroup and should not be generalized to all patients initiating the KM-OBP. Prospective multicenter research including non-responders and discontinuers and using validated acceptability measures is needed.

## Figures and Tables

**Table 1:** Patient satisfaction survey items.

Domain	Survey Item
Overall program evaluation	1. Overall, how satisfied are you with the treatment program? 2. What is the reason you were satisfied or dissatisfied overall?
Satisfaction with individual program components	3. How satisfied are you with the herbal medicine treatment? 4. How satisfied are you with the initial 3-day program? 5. How satisfied are you with dietary modification and nutritional counseling? 6. How satisfied are you with pharmacopuncture treatment? 7. How satisfied are you with thread embedding therapy? 8. How satisfied are you with radiofrequency treatment? 9. Please briefly explain the reason if you were dissatisfied with any of the program components.
Safety	10. How satisfied are you with the overall safety of the program of the clinic? 11. If you were not satisfied with the safety of the program, what was the reason?
Perceived effectiveness	12. Do you think the program was helpful in treating obesity? 13. If the program was not helpful, what was the reason?
Satisfaction with program-recommended lifestyle modifications	14. Did the program help you follow the lifestyle modifications recommended as part of the program, such as the low-carbohydrate diet? 15. If the program did not help you follow the recommended lifestyle modifications, such as the low-carbohydrate diet, what was the reason?
Recommendation and future intention	16. Would you recommend the program of the clinic to others? 17. Please explain the reason for recommending or not recommending the program. 18. Would you consider receiving treatment at a Korean medicine clinic again in the future if you develop other health conditions? 19. If you would not consider revisiting a Korean medicine clinic, what was the reason?
Treatment choice	20. Why did you choose Korean medicine for obesity treatment? 21. Why did you choose Daeat Clinic for treatment?

Items 1, 3–8, 10, 12, 14, 16, and 18 were rated on a 5-point Likert scale (1 = very dissatisfied; 2 = dissatisfied; 3 = neutral; 4 = satisfied; 5 = very satisfied). Items 2, 9, 11, 13, 15, 17, and 19 were optional explanatory free-text items. Items 20 and 21 were additional treatment-choice free-text prompts. Thus, nine free-text prompts were available for qualitative analysis: seven explanatory items and two treatment-choice items.

**Table 2:** Clinical characteristics of survey respondents and non-respondents.

	All Patients (*N* = 37)	Survey Respondents (*N* = 16)	Non-Respondents (*N* = 21)	SMD
	Mean (SD) or *n* (%)	Mean (SD) or *n* (%)	Mean (SD) or *n* (%)	
Age (years)	37.2 (10.7)	42.6 (11.8)	33.1 (7.8)	0.95
Sex				
Female	36 (97.3%)	15 (93.8%)	21 (100.0%)	−0.37
Male	1 (2.7%)	1 (6.2%)	0 (0.0%)	
BMI (kg/m^2^)	31.1 (1.10)	31.1 (0.92)	31.1 (1.24)	−0.09
Alcohol consumption	19 (51.4%)	9 (56.2%)	10 (47.6%)	0.17
Prior weight-loss treatment experience				
Conventional medicine	12 (32.4%)	7 (43.8%)	5 (23.8%)	0.43
Korean medicine	10 (27%)	5 (31.2%)	5 (23.8%)	0.17
Concomitant medications				
Hypertension	2 (5.4%)	2 (12.5%)	0 (0.0%)	0.53
Diabetes mellitus	2 (5.4%)	2 (12.5%)	0 (0.0%)	0.53
Dyslipidemia	1 (2.7%)	1 (6.2%)	0 (0.0%)	0.37
Thyroid diseases	2 (5.4%)	1 (6.2%)	1 (4.8%)	0.07
Neuropsychiatric diseases	5 (13.5%)	2 (12.5%)	3 (14.3%)	−0.05
Analgesics	1 (2.7%)	1 (6.2%)	0 (0.0%)	0.37
Concomitant symptoms				
Sleep disturbance	21 (56.8%)	9 (56.2%)	12 (57.1%)	−0.02
Peripheral edema	21 (56.8%)	9 (56.2%)	12 (57.1%)	−0.02
Difficulty with bowel movements	10 (27%)	6 (37.5%)	4 (19.0%)	0.42
Past medical history				
Allergic diseases	3 (8.1%)	2 (12.5%)	1 (4.8%)	0.28
Liver diseases	3 (8.1%)	1 (6.2%)	2 (9.5%)	−0.12
Pulmonary diseases	1 (2.7%)	1 (6.2%)	0 (0.0%)	0.37
Gastrointestinal diseases	14 (37.8%)	7 (43.8%)	7 (33.3%)	0.22
Genitourinary diseases	3 (8.1%)	2 (12.5%)	1 (4.8%)	0.28
Neuropsychiatric diseases	5 (13.5%)	1 (6.2%)	4 (19.0%)	−0.39
Family history				
Hypertension	3 (8.1%)	1 (6.2%)	2 (9.5%)	−0.12
Diabetes mellitus	3 (8.1%)	1 (6.2%)	2 (9.5%)	−0.12
Dyslipidemia	1 (2.7%)	1 (6.2%)	0 (0.0%)	0.37
Stroke	0 (0%)	0 (0%)	0 (0.0%)	0.00
Coronary disease	0 (0%)	0 (0%)	0 (0.0%)	0.00
Cancer	0 (0%)	0 (0%)	0 (0.0%)	0.00
Treatment duration, BMI measurement period (mean (SD); median (IQR)), and BMI change				
Treatment duration (months)	13.1 (5.8); 12 (9–15)	15.1 (5.0); 13.5 (12–19.3)	11.5 (5.9); 9 (9–12)	0.67
BMI change (kg/m^2^)	−8.9 (1.6)	−8.8 (1.5)	−9.0 (1.6)	0.18
BMI measurement period (months)	8.8 (2.5); 8 (7–10)	9.1 (3.2); 8.5 (7–11)	8.6 (1.8); 8 (8–10)	0.19
Time to ≥10% weight loss (months)	2.6 (1.3); 2 (2–3)	2.7 (1.6); 2 (2–3)	2.6 (1.0); 2 (2–3)	0.05
Time to BMI ≤ 23 kg/m^2^ (months)	7.8 (2.5)	8.5 (3.0)	7.2(2.0)	0.48
Any documented adverse event	25 (67.6%)	14 (87.5%)	11 (52.4%)	0.83

Values are presented as indicated. BMI, body mass index; IQR, interquartile range; SD, standard deviation; SMD, standardized mean difference. SMDs are descriptive and were not used for hypothesis testing. Treatment duration denotes participation in the KM-OBP; BMI measurement period denotes the interval from baseline to the last available BMI measurement.

**Table 3:** Domains, themes, subthemes, and illustrative quotes related to patient acceptability of the KM-OBP.

Domain	Theme	Subtheme	Illustrative Quote
Affective attitude	Positive emotional responses to treatment outcome and care	Successful weight loss, perceived health improvement, enhanced self-esteem	My appetite was well controlled without side effects, and I lost weight successfully, so overall I am very satisfied (P015-01).
		Trust in a structured program and professional expertise	The consultation was very helpful. I learned exactly what and how much to eat, and they helped me put it into practice in daily life. They clearly explained what should come first in dieting, which helped me stay consistent (P010-01).
	Negative emotional responses	Weight regains after program completion	I lost weight steadily, but after some time, the weight came back, which makes me very sad (P028-01).
		Perceived lack of proactive support	The so-called ‘close coaching’ only responds when I ask questions. It felt more like a Q&A than proactive consultation (P008-02).
Burden	Perceived financial burden	High cost	The cost feels like a heavy burden (P018-07).
	Burden of program adherence (dietary component)	Difficulty adhering to recommended diet	Because carbohydrates are restricted, it seems difficult for people who like carbs (P028-06).
Ethicality	Perception of safe weight loss	Expectation and experience of safety compared with conventional medications	I chose Korean medicine because I wanted to lose weight safely without side effects (P016-08).
		Monitoring, individualized care, and timely management of adverse events	When side effects like hair loss or skin trouble occurred, they responded immediately and managed it safely without major problems, which really reassured me (P021-03).
	Perceived price inequity	Concerns about price fairness and variability leading to reduced trust	Each staff member quoted a very different price. They said it depended on each person’s individual condition, but it felt unfair. After I pointed this out, they stopped sending me promotional messages (P029-06).
Intervention coherence	Understanding of program rationale and principles	Education on program principles, metabolism, and diet related to obesity and weight loss	They explained carbohydrates in detail, which helped me control my diet (P013-05).
Opportunity costs	Dietary trade-offs	Dietary restrictions perceived as manageable compared with other programs	I couldn’t eat everything, but compared to other diets, I could still eat to some extent, so it was manageable (P018-01).
		Greater difficulty for individuals who prefer carbohydrates	At first, many foods were restricted, so I thought anyone would lose weight like this. But after consciously reducing carbs with the medication, I kept some of the habits even when I stopped taking it (P002-05).
Perceived effectiveness	Perceived weight and body fat reduction	Achievement of weight loss with preferential reduction in body fat	When I first followed the diet advice, I lost muscle mass, but when I adjusted my approach, I lost body fat, which was good (P027-02). I was happy to lose mostly body fat (P017-04).
	Safety	Minimal side effects and perceived improvement in health indicators during herbal medicine use	With consistent management, my body wasn’t strained, so I felt it was safe (P028-03).
	Differential perceptions of program components	Individual variability in perceived effectiveness of adjunctive treatments	I’m not sure about the effects of devices or injections (P015-02). But for me, both herbal medicine and other treatments were effective, and after the initial 3-day program, I felt lighter (P007-02).
	Re-evaluation of effectiveness after program completion	Weight maintenance or regain after program completion	I didn’t experience severe rebound weight gain when I stopped the medication, and although there was a plateau, I lost a lot of weight after all (P002-01). But after the program ended, I started gaining some weight again (P018-04).
Self-efficacy	Enhanced capacity for sustained behavior change	Increased confidence through education, support, and individualized management	I was at a very low point, but participating in the program helped me a lot and boosted my confidence (P017-01). I changed through the program (P010-06)
	Post-program weight maintenance efforts	Commitment to ongoing lifestyle management	I need to manage myself continuously so that the weight doesn’t come back. (P017-07)

The opportunity-costs domain was represented by two participants and two coding units (Table A5); its illustrative quotations should therefore be interpreted as limited individual experiences.

**Table 4:** JOINT display integrating quantitative satisfaction ratings and qualitative findings.

Program Component	Quantitative Finding	Related Qualitative Finding	Integrated Interpretation
Overall program	High descriptive satisfaction (*n* = 16; mean 4.63)	Perceived weight-loss benefits, health improvement, structured delivery, and professional support; some accounts of weight regain	Predominantly convergent positive findings, with post-treatment weight regain adding complementary negative context
Herbal medicine	High descriptive satisfaction (*n* = 16; mean 4.56)	Perceived appetite control, effectiveness, safety, and individualized prescribing	Convergence between ratings and positive experiences; perceived effectiveness was not interpreted as demonstrated efficacy
Dietary modification and nutritional counseling	More variable ratings (*n* = 13; mean 3.92; range 1–5)	Carbohydrate restriction and difficulty maintaining dietary changes were described as burdens	Complementarity: qualitative accounts explained heterogeneity in the descriptive ratings
Pharmacopuncture	Lower descriptive rating among exposed respondents (*n* = 11; mean 3.64)	Some respondents expressed uncertainty about the effects of injections or devices	Convergence between comparatively lower ratings and uncertainty, interpreted cautiously because of the small denominator

Quantitative ratings are descriptive. Item denominators differ because patients not exposed to an optional modality were excluded as not applicable. Qualitative findings provide experiential context and do not establish treatment efficacy.

## Data Availability

The data supporting the findings of this study are not publicly available. De-identified data may be made available from the corresponding authors upon reasonable request and with appropriate institutional approval.

## Abbreviations

The following abbreviations are used in this manuscript:

KM-OBP	Korean medicine-based obesity treatment program
BMI	Body mass index
KM	Korean medicine
SDs	Standard deviations

## Appendix A

### Appendix A.1. Korean Medicine-Based Obesity Treatment Program Implemented at Daeat Korean Medicine Clinic

#### Appendix A.1.1. Initial 3-Day Program

The initial 3-day program was designed to promote appetite suppression and early weight loss through Haedok-dan, to facilitate gastrointestinal function and bowel movements through Bium-dan, and to achieve caloric restriction while increasing intake of complex carbohydrates by restricting regular meals and providing Daeat Bium Enzyme and Daeat Balance.

(1) Haedok-dan

Haedok-dan (Table A1) is an herbal medicine prescribed primarily for weight loss and induction of satiety, with Ephedrae Herba as its main ingredient. Patients took one sachet three times daily for three consecutive days.

(2) Bium-dan

Bium-dan (Table A1) is an herbal formulation composed of purgative herbs, including Rhei Radix et Rhizoma, and was prescribed to promote bowel movements. Patients took one sachet per day for three days, with administration and dosage adjusted according to bowel movement status based on medical staff instructions.

**Table A1:** Composition of Haedok-dan and Bium-dan used in the initial 3-day program.

Prescription	Scientific Name	Pinyin Name (Chinese Name)	Herbal Name	Single Dose (g)
Haedok-dan (5.0 g/ea)				
	*Ephedra sinica* Stapf	Mahuang (麻黃)	Ephedrae Herba	1.25
	*Coix lacryma-jobi* L.	Yiyiren (薏苡仁)	Coicis Semen	0.94
	*Rehmannia glutinosa* (Gaertn.) Libosch. ex DC.	Shudihuang (熟地黃)	Rehmanniae Radix Preparata	0.94
		Shigao (石膏)	Gypsum Fibrosum	0.63
	*Poria cocos* (Schw.) Wolf	Fuling (茯苓)	Poria Sclerotium	0.63
	*Senna alexandrina* var. *alexandrina*	Fanxieye (番瀉葉)	Sennae Folium	0.31
	*Atractylodes lancea* (Thunb.) DC.	Cangzhu (蒼朮)	Atractylodis Rhizoma	0.31
Bium-dan (5.0 g/ea)				
	*Rheum officinale* Baill.	Dahuang (大黃)	Rhei Radix et Rhizoma	1.5
	*Senna alexandrina* var. *alexandrina*	Fanxieye (番瀉葉)	Sennae Folium	1.5
	*Angelica sinensis* (Oliv.) Diels	Danggui (當歸)	Angelicae Sinensis Radix	0.5
	*Prunus humilis* Bunge	Yuliren (郁李仁)	Pruni Japonicae Semen	0.5
	*Prunus persica* Batsch	Taoren (桃仁)	Persicae Semen	0.5
	*Prunus armeniaca* L.	Xingren (杏仁)	Armeniacae Semen	0.5

(3) Daeat Bium Enzyme

Daeat Bium Enzyme (METAPHARM Co., Ltd., Seoul, Republic of Korea) is a dietary supplement prescribed as an adjunct during obesity treatment to support digestive function. Patients consumed one sachet (3.0 g) per day for three days, taken either directly or mixed with water. The product contains an enzyme-based food (NPK Foodzyme E4) comprising 50% of the formulation, which includes fermented mixed grains, NPK fermented enzyme powder 3 (oats and brown rice), potato extract powder, and papaya extract powder. Other main ingredients include alpha brown rice powder, red soybean powder, crystalline fructose, bamboo leaf extract powder, roasted rice cake flavor powder (artificial flavoring), enzyme-treated stevia, rice protein powder, chicory root extract, psyllium husk powder, a vitamin–mineral premix (dicalcium phosphate, vitamin C, ferrous fumarate, vitamin E, nicotinamide, zinc oxide, vitamin A, pyridoxine hydrochloride, thiamine hydrochloride, riboflavin, and folic acid), a mixed probiotic powder, an amino acid blend (L-methionine, L-tryptophan, L-glutamine, L-proline, and glycine), fish collagen, and a hyaluronic acid blend (dextrin and hyaluronic acid). The product contains milk and soy.

(4) Daeat Balance

Daeat Balance (METAPHARM Co., Ltd., Seoul, Republic of Korea) is a meal-replacement product developed for use during the initial 3-day program and contains carbohydrates and plant-based protein. Each sachet contains 10 g of carbohydrates, resulting in a total daily carbohydrate intake of 30 g when three sachets are consumed per day. The main ingredients include soy protein, whey protein isolate powder, mixed grain powder, erythritol, pea protein isolate, roasted soybean powder mix (SR-28837), crunchy granola, polydextrose, enzyme-treated stevia, yeast (MNY), glucomannan, marine-derived calcium, psyllium husk, organic freeze-dried mixed root vegetable powder (11 varieties), organic freeze-dried black food blend powder, amino acid mix, and mixed grain fermented enzymes.

#### Appendix A.1.2. Treatment Program Components Following the Initial 3-Day Program

Herbal medicine prescriptions are determined by the attending Korean medicine physician based on each patient’s clinical characteristics and formulation preferences. The core herbal medicine formulation used in the program is presented in Table A2. The core formulation is composed of herbs intended to primarily promote appetite suppression, weight loss, enhancement of satiety, activation of gastrointestinal function, and support the elimination of metabolic waste. The formulations are prescribed in various dosage forms, including pills, tablets, capsules, or decoctions, according to patient preferences, with selected herbs added or omitted based on individual clinical circumstances. Throughout the entire program period, all participants adjust their diet in accordance with instructions provided by Korean medicine physicians. After the initial 3-day program, the dietary component of the program is based on a low-carbohydrate approach, with additional intake of small amounts of high-protein foods, such as boiled eggs or tofu, recommended during periods of hunger. To ensure safe obesity treatment, adequate fluid intake (approximately 1.5–2.5 L per day for the average adult) is encouraged to maintain hydration and electrolyte balance, along with avoidance of excessive potassium intake, while sodium intake is not specifically restricted. Patients are advised to maintain light, routine physical activity. In addition, concomitant use of agents with diuretic or laxative effects is discouraged, and patients are instructed to report any adverse events to the medical staff promptly.

**Table A2:** The core herbal medicine formulations prescribed after the initial 3-day program.

Scientific Name	Pinyin Name (Chinese Name)	Herbal Name	Daily Dose (g)
*Ephedra sinica* Stapf	Mahuang (麻黄)	Ephedrae Herba	7.50–18.00 *
*Coix lacryma-jobi* var. *ma-yuen* (Rom.Caill.) Stapf	Yiyiren (薏苡仁)	Coicis Semen	0.90
*Artemisia capillaris* Thunb.	Yinchenhao (茵蔯蒿)	Artemisiae Capillaris Herba	0.90
*Gardenia jasminoides* J. Ellis	Zhizi (栀子)	Gardeniae Fructus	0.90
*Areca catechu* L.	Binlang (檳榔)	Arecae Semen	0.45
*Rehmannia glutinosa* (Gaertn.) DC. (processed root)	Shudihuang (熟地黃)	Rehmanniae Radix Preparata	0.45
*Pueraria lobata* (Willd.) Ohwi	Gegen (葛根)	Puerariae Radix	0.45
*Atractylodes macrocephala* Koidz.	Baizhu (白术)	Atractylodis Rhizoma Alba	0.45
*Zingiber officinale* Roscoe	Shengjiang (生姜)	Zingiberis Rhizoma Recens	0.45
*Poria cocos* (Schw.) Wolf	Fuling (茯苓)	Poria Sclerotium	0.45
*Citrus unshiu* Markov.	Chenpi (陳皮)	Citri Unshius Pericarpium	0.45
*Magnolia officinalis* Rehder & E.H. Wilson	Houpo (厚朴)	Magnoliae Cortex	0.45
*Poncirus trifoliata* (L.) Raf.	Zhishi (枳実)	Ponciri Fructus Immaturus	0.45
*Caesalpinia sappan* L.	Sumu (蘇木)	Sappan Lignum	0.45

*, Herbal medicine formulations prescribed after the initial 3-day program. The ephedra content per sachet is divided into 6 levels: 2.5 g, 3.2 g, 3.9 g, 4.6 g, 5.3 g, and 6 g.

**Table A3:** Adverse events documented during the Korean medicine-based obesity treatment program.

	All Patients (*n* = 37)	Survey Respondents (*n* = 16)	Non-Respondents (*n* = 21)
	*n* (%)	*n* (%)	*n* (%)
Constipation	20 (54.1)	12 (75.0)	8 (38.1)
Heartburn	2 (5.4)	0 (0)	2 (9.5)
Nausea	7 (18.9)	6 (37.5)	1 (4.8)
Acid regurgitation	2 (5.4)	1 (6.3)	1 (4.8)
Diarrhea	1 (2.7)	0 (0)	1 (4.8)
Dry mouth	6 (16.2)	5 (31.3)	1 (4.8)
Insomnia	4 (10.8)	2 (12.5)	2 (9.5)
Tremor	1 (2.7)	1 (6.3)	0 (0.0)
Dizziness	7 (18.9)	4 (25.0)	3 (14.3)
Headache	1 (2.7)	0 (0)	1 (4.8)
Fatigue	3 (8.1)	1 (6.3)	2 (9.5)
Leg edema	2 (5.4)	1 (6.3)	1 (4.8)
Chest discomfort	2 (5.4)	0 (0)	2 (9.5)
Palpitations	1 (2.7)	1 (6.3)	0 (0.0)
Myalgia	1 (2.7)	1 (6.3)	0 (0.0)
Alopecia	1 (2.7)	1 (6.3)	0 (0.0)
Urticaria	1 (2.7)	1 (6.3)	0 (0.0)
Menstrual irregularity	1 (2.7)	0 (0)	1 (4.8)

Values are *n* (%), representing patients with at least one documented occurrence of each adverse event during treatment. Individual patients could contribute to more than one category. Severity was not formally graded.

**Table A4:** Patient satisfaction summary statistics and response distributions on a 5-point Likert scale.

Patient Satisfaction Survey Items	*N*	Mean (SD)	Median (Q1, Q3)	Min–Max	Scale 1 *n* (%)	Scale 2 *n* (%)	Scale 3 *n* (%)	Scale 4 *n* (%)	Scale 5 *n* (%)
Satisfaction with overall treatments	16	4.63 (0.62)	5 (4, 5)	3–5	0 (0)	0 (0)	1 (6.3)	4 (25.0)	11 (68.8)
Satisfaction with herbal medicine	16	4.56 (0.51)	5 (4, 5)	4–5	0 (0)	0 (0)	0 (0)	7 (43.8)	9 (56.3)
Satisfaction with initial 3-day program	14	4.07 (1)	4 (3.25, 5)	2–5	0 (0)	1 (7.1)	3 (21.4)	4 (28.6)	6 (42.9)
Satisfaction with dietary modification and nutritional counseling	13	3.92 (1.32)	4 (3, 5)	1–5	1 (7.7)	1 (7.7)	2 (15.4)	3 (23.1)	6 (46.2)
Satisfaction with pharmacopuncture	11	3.64 (0.81)	3 (3, 4)	3–5	0 (0)	0 (0)	6 (54.5)	3 (27.3)	2 (18.2)
Satisfaction with thread embedding therapy	7	4.43 (0.79)	5 (4, 5)	3–5	0 (0)	0 (0)	1 (14.3)	2 (28.6)	4 (57.1)
Satisfaction with radiofrequency treatment	11	3.91 (0.83)	4 (3, 4.5)	3–5	0 (0)	0 (0)	4 (36.4)	4 (36.4)	3 (27.3)
Satisfaction with safety	16	4.38 (0.72)	4.5 (4, 5)	3–5	0 (0)	0 (0)	2 (12.5)	6 (37.5)	8 (50.0)
Helpful in treating obesity	16	4.63 (0.62)	5 (4, 5)	3–5	0 (0)	0 (0)	1 (6.3)	4 (25.0)	11 (68.8)
Recommendation intention	16	4.38 (0.81)	5 (4, 5)	3–5	0 (0)	0 (0)	3 (18.8)	4 (25.0)	9 (56.3)
Intention to revisit Korean medicine clinics for other diseases	16	3.81 (1.17)	4 (3.5, 5)	2–5	0 (0)	4 (25.0)	0 (0)	7 (43.8)	5 (31.3)

A 5-point Likert scale was used: 5, very satisfied; 4, satisfied; 3, neutral; 2, dissatisfied; 1, very dissatisfied. Values in the distribution panel are *n* (%). Scale 1–Scale 5 indicate the number and percentage of respondents who selected each response option on the five-point Likert scale for each survey item. Different denominators reflect patients who were not exposed to the corresponding optional modality and were treated as not applicable rather than missing.

**Table A5:** Extent and participant contribution of the free-text qualitative dataset.

Panel	Item/Domain	Respondents, *n* (%)	Responses/Coding Units, *n*
Prompt-level responses			
	Overall satisfaction reason	14 (87.5)	14
	Component evaluation reason	11 (68.8)	11
	Safety reason	8 (50.0)	8
	Obesity-treatment helpfulness reason	12 (75.0)	12
	Lifestyle-modification helpfulness reason	10 (62.5)	10
	Recommendation reason	13 (81.2)	13
	Future KM-treatment intention reason	9 (56.2)	9
	Reason for choosing Korean medicine	15 (93.8)	15
	Reason for choosing Daeat Clinic	15 (93.8)	15
TFA-domain contribution			
	Burden	16 (100.0)	33
	Affective attitude	15 (93.8)	33
	Ethicality	15 (93.8)	35
	Perceived effectiveness	13 (81.3)	25
	Intervention coherence	11 (68.8)	21
	Self-efficacy	9 (56.3)	15
	Opportunity costs	2 (12.5)	2

All 16 survey respondents provided at least one free-text response. The fixed dataset comprised 107 responses, 140 sentences, and 931 words across seven explanatory prompts and two treatment-choice prompts. A response could receive more than one code; coding-unit totals therefore exceed response totals.
